# Relative Deprivation, Income Inequality, and Cardiovascular Health: Observational and Mendelian Randomization Studies in Hong Kong Chinese

**DOI:** 10.3389/fpubh.2021.726617

**Published:** 2022-01-21

**Authors:** Man Ki Kwok, Ichiro Kawachi, David Rehkopf, Michael Y. Ni, Gabriel M. Leung, C. Mary Schooling

**Affiliations:** ^1^School of Public Health, Li Ka Shing Faculty of Medicine, The University of Hong Kong, Hong Kong, Hong Kong SAR, China; ^2^Department of Social and Behavioral Sciences, Harvard T. H. Chan School of Public Health, Harvard University, Boston, MA, United States; ^3^Department of Epidemiology and Population Health, Stanford University, Stanford, CA, United States; ^4^Healthy High Density Cities Lab, HKUrbanLab, The University of Hong Kong, Hong Kong, Hong Kong SAR, China; ^5^The State Key Laboratory of Brain and Cognitive Sciences, The University of Hong Kong, Hong Kong, Hong Kong SAR, China; ^6^City University of New York Graduate School of Public Health and Health Policy, New York, NY, United States

**Keywords:** adult, absolute income, relative income, cortisol, cardiovascular disease, Mendelian randomization

## Abstract

The associations between absolute vs. relative income at the household or neighborhood level and cardiovascular disease (CVD) risk remain understudied in the Chinese context. Further, it is unclear whether stress biomarkers, such as cortisol, are on the pathway from income to CVD risk. We examined the associations of absolute and relative income with CVD risk observationally, as well as the mediating role of cortisol, and validated the role of cortisol using Mendelian Randomization (MR) in Hong Kong Chinese. Within Hong Kong's FAMILY Cohort, associations of absolute and relative income at both the individual and neighborhood levels with CVD risk [body mass index (BMI), body fat percentage, systolic blood pressure, diastolic blood pressure, self-reported CVD and self-reported diabetes] were examined using multilevel logistic or linear models (*n* = 17,607), the mediating role of cortisol using the mediation analysis (*n* = 1,562), and associations of genetically predicted cortisol with CVD risk using the multiplicative generalized method of moments (MGMMs) or two-stage least squares regression (*n* = 1,562). In our cross-sectional observational analysis, relative household income deprivation (per 1 SD, equivalent to USD 128 difference in Yitzhaki index) was associated with higher systolic blood pressure (0.47 mmHg, 95% CI 0.30–0.64), but lower BMI (−0.07 kg/m^2^, 95% CI −0.11 to −0.04), independent of absolute income. Neighborhood income inequality was generally unrelated to CVD and its risk factors, nor was absolute income at the household or neighborhood level. Cortisol did not clearly mediate the association of relative household income deprivation with systolic blood pressure. Using MR, cortisol was unrelated to CVD risk. Based on our findings, relative household income deprivation was not consistently associated with cardiovascular health in Hong Kong Chinese, nor were neighborhood income inequality and absolute income, highlighting the context-specific ways in which relative and absolute income are linked to CVD risk.

## Introduction

Economic growth in China has been accompanied by rising income inequality (1). In previous studies, income inequality was linked to the increased risks of cardiovascular disease (CVD) mortality (2) as well as risk factors (such as, high blood pressure and overweight) in the United States (3, 4), though not consistently in Europe (5–7) or New Zealand (8).

A concept related to income inequality is relative income deprivation (9). Growing inequality in the distribution of incomes will tend to widen the gap between the incomes of individuals, triggering stressful social comparisons, and an increased sense of *relative* income deprivation. Notably, the harmful effects of relative income deprivation can occur even in the absence of absolute income deprivation. For example, an individual may not be deprived in an absolute sense (i.e., an individual has sufficient income to satisfy the need for food, clothing, and shelter), and yet feel relatively deprived when one perceives that other people are overtaking, as income inequality widens. In turn, a growing sense of relative deprivation is hypothesized to be linked to the psychosocial stress, as well as maladaptive coping responses to stress, e.g., smoking, excess drinking, and binge snacking (10). Stress due to upward social comparisons may also give rise to more negative emotions (such as, anger or depression), which are in turn correlated with the higher blood pressure (11).

Relative income deprivation has been linked with ischemic heart disease (IHD) mortality and/or incidence in the United States (12), the United Kingdom (13), and Japan (14). In China, most of the previous studies considered the effects of income and income disparity separately, with inconsistent findings for CVD and its risk factors (15–18). A Chinese study that examined both absolute and relative income found that income inequality is inversely associated with overweight but also with higher waist circumference in older adults (19). In addition, it found relative income deprivation to be positively correlated with hypertension in urban areas, but also inversely associated with overweight and waist circumference (19).

To assess whether absolute or relative income affects cardiovascular health, and to clarify the relevance of the mechanism of cortisol, we used two complementary designs in Hong Kong, a rapidly developed Chinese setting with a wide income disparity (Gini = 0.539). First, using an observational study, we examined the associations of absolute and relative household and neighborhood income with CVD and its risk factors in Hong Kong Chinese. Besides, we examined whether the association of relative household income deprivation with CVD and its risk factors was mediated by cortisol, a potential stress mechanism. Second, using a one-sample Mendelian randomization (MR) study, which is less prone to confounding due to proxying exposures by randomly assigned genetic variation (20), we assessed whether cortisol was associated with CVD and its risk factors in Hong Kong Chinese.

## Materials and Methods

### Data Sources

#### Observational and MR Studies in Chinese

The FAMILY Cohort is a population-based household study in Hong Kong recruited from March 2009 to March 2014, as detailed elsewhere (21). It aims to promote individual and household health, happiness, and family harmony (3Hs). A family living in the same household was used as the sampling unit, with all family members aged ≥10 years were eligible to participate. Based on the stratified random sampling, residential addresses were sampled in 18 Hong Kong districts, 19,533 Chinese participants (from 8,115 households) were recruited as the random core sample. The study further recruited first-degree relatives of this sample and oversampled three new towns, population subgroups, and certain districts, comprising 46,001 Chinese participants (from 20,279 households) as the entire sample. Two household visits and five telephone- or web-based follow-ups were conducted in 2009–2014. The entire sample completed the first household visit (wave 1) in March 2009 to April 2011, and those aged ≥ 15 years were followed-up in the second household visit (wave 2) in August 2011 to March 2014. During household visits, eligible participants were asked to complete an interviewer-administered survey covering information on socio-demographics, lifestyle, and behavioral factors, health indicators and standardized instruments assessing health, and happiness and family harmony (22). Anthropometrics, body fat percentage, and blood pressure were measured by the interviewers. Height was measured by a Mobile Stadiometer Seca 214, USA; weight and body fat percentage were measured by a Body Fat Analyzer Scale Omron HBF-356 (Japan); blood pressure, measured using an Omron HEM-7000, was the average of two measurements taken after at least 5 min of rest.

A Biobank in the FAMILY Cohort was established in May 2016, targeting participants aged ≥ 8 years living in two districts (Tin Shui Wai and Sham Shui Po). During the follow-up, non-fasting blood samples were collected in the morning or afternoon and then stored at −80°C. For cortisol, serum samples were tested in an accredited laboratory (PathLab Medical Laboratories, HK). Deoxyribonucleic acid (DNA) extraction from whole blood and MassARRAY genotyping were conducted at the Centre for PanorOmic Sciences of the University of Hong Kong. The DNA samples were analyzed using iPLEX MassARRAY system for genotyping candidate single nucleotide polymorphisms (SNPs) related to cortisol. Ethical approval for this sub-study was obtained from the University of Hong Kong/Hospital Authority Hong Kong West Cluster Institutional Review Board. Informed written consent was obtained from the participants before participation.

### Exposures

#### Observational Study in the FAMILY Cohort

##### Household Income

Monthly average household income before tax (including all income sources and compulsory pension fund, i.e., Mandatory Provident Fund) reported by the head of household (defined as the main income earner within the family) in wave 1 was recorded in 29 income categories (ranging from HK$1-$499 to HK$150,000 or above). It was divided by the square root of household size (an equivalized on a scale of 0.5) to account for the proportional needs for shared commodities (23).

##### Neighborhood Income

Hong Kong is divided into small neighborhoods of residence called Tertiary Planning Units (TPUs) which represent clustered geographical units created for town planning purposes. Based on the 2011 Population Census in Hong Kong, there were 289 TPUs for which median monthly domestic household income was available. Each participant from the FAMILY Cohort was assigned to a TPU, and the associated neighborhood income, based on anonymous addresses recorded in wave 1.

Household income and neighborhood income were mapped to absolute and relative income constructs as follows:

- *Absolute household income*: equivalized monthly average household income.
- *Absolute neighborhood median income*: TPU median monthly household income in quantiles (from lowest to highest) (24).
- *Relative household income deprivation*: measured by the Yitzhaki index (25), i.e., average difference in the equivalized household income between the index household and every other household with a higher household income in the comparison group (within the FAMILY Cohort).
- *Neighborhood income inequality*: proxied by a TPU-specific Gini coefficient, a widely used indicator of income inequality ranging from 0 (perfect equality) to 1 (perfect inequality) in quantiles (from the most similar to the widest income distribution) (24).

### Outcomes

#### CVD and Its Risk Factors

Cardiovascular disease was based on the self-reported coronary heart disease, stroke, or other heart disease in wave 1. CVD risk factors were based on the self-reported diabetes, body mass index (BMI), body fat percentage, and systolic and diastolic blood pressure measured in wave 1. BMI was calculated from weight divided by square of height.

### Statistical Analysis

For the observational study, we examined the associations of the four income measures with CVD and its risk factors (BMI, body fat percentage, and blood pressure, self-reported CVD, and diabetes) using a fixed effects multilevel linear or logistic model. The beta coefficient (mean difference) or odds ratio (OR) with 95% CI for mutually adjusted associations accounting for individuals being nested within families and neighborhoods using intraclass correlations (variation within and between these groups), adjusted for potential confounders (age, sex, and birthplace) is presented. While some other confounders, such as level of education, may exist for individual level income exposures, we used this more parsimonious set of potential confounders to fit consistent models across the household and neighborhood level income exposures we examined.

In the mediation analysis, to obtain direct and indirect effects and the proportion mediated, we assessed the association of relative household income deprivation (based on equivalized household income reported in the Biobank follow-up) with CVD and its risk factors, such as cortisol, the interaction of cortisol with relative household income deprivation, and the potential confounders (age, sex, and birthplace).

## MR Study in the FAMILY Cohort

### Exposures

#### Selection of Candidate SNPs

To identify genetic instrument of cortisol in the Chinese whom a genome-wide association study (GWAS) for cortisol is unavailable, we initially selected the 539 non-identical (*R*^2^ < 1), biallelic SNPs suggestively associated with cortisol (*P* < 1 × 10^−4^) in any of the three existing cortisol GWAS in people of European descent (26–28) with minor allele frequency (MAF) in Chinese ≥ 0.05 based on 1,000 Genomes East Asian allele frequency. We further excluded correlated SNPs (*R*^2^ ≥ 0.05) and SNPs that failed to assay design restricted to 3 multiplex assay groups and focused on SNPs with MAF > 0.30 in Chinese, giving 84 SNPs.

After genotyping, quality control procedures were carried out by excluding samples with (1) SNP call rate < 0.02; (2) sample call rate < 0.02; (3) MAF < 0.05; (4) Hardy–Weinberg equilibrium *p* < 1 × 10^−6^; (5) sample heterozygosity rate outliers (mean ± 3 SD). Principal components were estimated based on the remaining 61 independent (*r*^2^ < 0.01) SNPs (29).

#### Genetic Instrument for Cortisol

We used an internally weighted polygenic score (PGS) for log-transformed cortisol aligned on the cortisol increasing allele. The PRS was calculated by multiplying the genotype with internal weights, taken from the generalized least squares method accounting for kinship matrix (sample relatedness), adjusted for age, sex, and the first 10 principal components.

### Statistical Analysis

We obtained beta coefficients and 95% CIs for the association of the cortisol PRS with BMI, body fat percentage, and blood pressure using the 2-stage least squares (2SLS). We obtained ORs for the association of the cortisol PRS with CVD and diabetes using the multiplicative generalized method of moments (MGMMs) (30) with bootstrapping with 1,000 replications to estimate CIs, given the variance of the error terms was heteroskedastic. We used the Hausman test to assess whether the 2SLS estimates differed from the linear regression estimates adjusted for age and sex and whether the MGMM estimates differed from the Poisson regression estimates adjusted for age and sex to which MGMM is equivalent.

Statistical analyses were conducted using Stata version 15.1 (Stata Corp, College station, TX, USA), and R version 4.0.3 (R Foundation for Statistical Computing, Vienna, Austria) with the statgenGWAS, MendelianRandomization, and MRPRESSO R packages, and PLINK version 1.9 (http://pngu.mgh.harvard.edu/purcell/plink/) unless specified. To account for multiple comparisons, we used a Bonferroni correction (*p* < 0.001, i.e., 0.05/48).

## Results

### Observational Study

A total of 17,607 Chinese adults were included in the analysis, with more than half being women and born in Hong Kong (Table 1). The average age was 44.0 years (SD 20.3). The equivalized monthly average household income was HK$ 15,712 (SD 20,256) and the relative income deprivation was HK$ 8,685 (SD 3,792), i.e., on average, a household had HK$ 8,685 less income than higher-income households. The mean BMI was 23.2 kg/m^2^, mean body fat percentage was 27.0%, and mean systolic/diastolic blood pressure was 125/78 mmHg. CVD was reported by 4.6% and diabetes by 6.5%.

**Table 1:** Baseline characteristics for 17,607 Hong Kong Chinese adults in the observational study of income and cardiovascular health and for 1,562 Chinese adults in the mediation analysis and Mendelian randomization study of cortisol and cardiovascular health from Hong Kong's FAMILY Cohort.

**Characteristics**	**Observational study** ^**^†^**^			**Mediation analysis and**		
	**(*****n*** **=** **17,607)**			**Mendelian randomization**		
				**(*****n*** **=** **1,562)**		
	**No**.	**%**	**Mean (SD)**	**No**.	**%**	**Mean (SD)**
**Sex**						
Men	8,283	47.0		671	43.7	
Women	9,324	53.0		865	56.3	
**Age** (years)	17,607		44.0 (20.3)	1,536		44.9 (16.2)
**Place of birth**						
Hong Kong	8,732	53.9		816	54.8	
Mainland China or elsewhere	7,484	46.2		672	45.2	
**Highest education**						
Primary	4,594	32.8		320	24.5	
Lower secondary	3,234	23.1		339	26.0	
Upper secondary	3,717	26.6		381	29.2	
Tertiary (non-degree)	1,208	8.6		124	9.5	
Tertiary (degree or above)	1,242	8.9		140	10.7	
**Absolute household income**			15,712 (20,256)			
**Relative household income deprivation**			8,685 (3,792)			10,895 (4,860)
**Absolute neighborhood median income**						
1st	4,626	26.3				
2nd	4,795	27.2				
3rd	4,402	25.0				
4th	3,784	21.5				
**Neighborhood income inequality**						
1st	5,082	28.9				
2nd	3,960	22.5				
3rd	4,291	24.4				
4th	4,274	24.3				
**Self-reported cardiovascular disease**						
No	15,370	95.4		1,423	96.4	
Yes	734	4.6		53	3.6	
**Self-reported diabetes**						
No	15,052	93.5		1,406	95.3	
Yes	1,052	6.5		70	4.7	
**Body mass index** (kg/m^2^)	17,374		23.2 (4.0)	1,516		23.5 (3.8)
**Body fat percentage** (%)	13,927		27.0 (7.4)	1,379		27.3 (6.8)
**Systolic blood pressure** (mmHg)	17,444		125.6 (21.2)	1,533		123.7 (19.2)
**Diastolic blood pressure** (mmHg)	17,432		78.3 (11.8)	1,531		78.0 (11.3)
**Cortisol** (ug/dl)				1,562		8.2 (3.3)
**Log-transformed cortisol**				1,562		2.0 (0.4)

† *Absolute household income and relative household income deprivation were based on equivalized monthly household income per head in Hong Kong dollar pegged at a rate of 7.8 dollar = 1 U.S. dollar*. *Absolute neighborhood median income in Hong Kong dollar (mean; range): 1st quartile ($14,213; $8,000–15,400), 2nd quartile ($17,350; $15,500–19,000), 3rd quartile ($20,436; $190,40–22,000), and 4th quartile ($22,040; $22,040–99,000)*. *Neighborhood income inequality in Gini (mean; range): 1st quartile (0.39; 0.21–0.40), 2nd quartile (0.42; 0.40–0.43), 3rd quartile (0.43; 0.43–0.44), and 4th quartile (0.46; 0.44–0.51)*.

Table 2 shows the adjusted associations of income with CVD and its risk factors. When all income measures were considered together, after accounting for multiple comparisons, greater relative household income deprivation was associated with lower BMI but not with body fat percentage, as well as higher systolic but not diastolic blood pressure. Relative income deprivation was not associated with CVD or diabetes. Greater neighborhood income inequality (4th quartile) was associated with lower BMI, after accounting for multiple comparisons. Higher absolute household income was associated with higher body fat percentage and higher absolute neighborhood median income (4th quartile) was associated with lower blood pressure at a nominal level of statistical significance, but not after accounting for multiple comparisons. There were no other associations for absolute income at the household or neighborhood level, nor for the neighborhood income inequality.

**Table 2:** Adjusted^†^ association of absolute household income, absolute neighborhood median income, relative household income deprivation, and neighborhood income inequality with cardiovascular disease (CVD) risk factors [body mass index (BMI), body fat percentage, and systolic and diastolic blood pressure] as well as self-reported CVD and diabetes for 17,607 Hong Kong Chinese adults from Hong Kong's FAMILY Cohort.

	**Body mass index**		**Body fat percentage**		**Systolic blood pressure**		**Diastolic blood pressure**		**Self-reported cardiovascular disease**		**Self-reported diabetes**	
	**β**	**95% CI**	**β**	**95% CI**	**β**	**95% CI**	**β**	**95% CI**	**OR**	**95% CI**	**OR**	**95% CI**
**Absolute household income** (per HKD 1,000 or USD 128)	−0.004	−0.01, 0.002	0.01	0.004, 0.02	0.01	−0.02, 0.04	−0.02	−0.04, 0.00	1.01	1.00, 1.02	1	0.99, 1.01
**Absolute neighborhood median income**												
1st quartile	Reference		Reference		Reference		Reference		1		1	
2nd quartile	0.09	−0.10, 0.27	0.18	−0.20, 0.56	−0.27	−1.12, 0.59	−0.22	−0.84, 0.40	0.96	0.77, 1.20	0.96	0.78, 1.18
3rd quartile	0.03	−0.16, 0.22	0.04	−0.33, 0.42	−0.15	−1.03, 0.72	0.07	−0.55, 0.70	0.81	0.64, 1.02	0.97	0.79, 1.20
4th quartile	−0.17	−0.38, 0.03	0.05	−0.33, 0.44	−1.59	−2.55, −0.64	−0.91	−1.57, −0.24	0.89	0.69, 1.15	0.95	0.75, 1.20
**Relative household income deprivation** (per HKD 1,000 or USD 128)	**−0.07**	−0.11, −0.04	0.01	−0.04, 0.07	**0.47**	0.30, 0.64	−0.07	−0.18, 0.03	1.07	1.02, 1.12	1.01	0.97, 1.05
**Neighborhood income inequality**												
1st quartile	Reference		Reference		Reference		Reference		1		1	
2nd quartile	−0.25	−0.45, −0.06	−0.27	−0.66, 0.12	−0.20	−1.11, 0.71	0.17	−0.48, 0.82	0.94	0.72, 1.21	0.88	0.71, 1.11
3rd quartile	−0.12	−0.31, 0.07	−0.19	−0.59, 0.20	−0.49	−1.37, 0.40	−0.16	−0.81, 0.48	0.94	0.73, 1.20	0.98	0.78, 1.23
4th quartile	**−0.33**	−0.51, −0.14	−0.16	−0.53, 0.20	−0.18	−1.05, 0.69	−0.27	−0.89, 0.35	1.24	0.98, 1.57	0.84	0.68, 1.04
Total no.	16,035		13,907		16,073		16,065		16,081		16,081	

*CI, confidence interval; OR, odds ratio; β, beta coefficient (mean difference)*.

*Bold indicates statistical significance after accounting for multiple comparisons using Bonferroni correction (p < 0.001)*.

† *Multilevel linear or logistic regression with absolute household income, absolute neighborhood median income, relative household income deprivation, and neighborhood income inequality adjusted for age, sex, and birthplace*.

Table 3 shows the adjusted mediation analysis in those with cortisol measurement (*n* = 1,562). Cortisol partially mediated the positive association of relative household income deprivation with systolic blood pressure at a nominal level of statistical significance, but not after accounting for multiple comparisons, whereas it did not mediate the association with diastolic blood pressure. The relative household income deprivation was not associated with BMI, body fat percentage, self-reported CVD, or diabetes.

**Table 3:** Adjusted^†^ total effect, direct effect of relative household income deprivation (effect not *via* mediator) and indirect effect of cortisol (mediator) with CVD risk factors (BMI, body fat percentage, and systolic and diastolic blood pressure) as well as self-reported CVD and diabetes^*^ for 1,562 Hong Kong Chinese adults from Hong Kong's FAMILY Cohort.

	**Body mass index**		**Body fat percentage**		**Systolic blood pressure**		**Diastolic blood pressure**		**Self-reported cardiovascular disease**		**Self-reported diabetes**	
	**β**	**95% CI**	**β**	**95% CI**	**β**	**95% CI**	**β**	**95% CI**	**Estimate**	**95% CI**	**Estimate**	**95% CI**
Indirect effect	−0.01	−0.01, 0.00	−0.005	−0.01, 0.00	0.01	0.001, 0.04	0.01	−0.003, 0.02	0.00004	−0.0001, 0.00	0.00007	−0.00001, 0.00
Direct effect	0.004	−0.04, 0.04	−0.01	−0.07, 0.04	**0.35**	0.16, 0.53	0.13	0.01, 0.25	0.0004	−0.002, 0.00	0.001	−0.0001, 0.00
Total effect	−0.002	−0.04, 0.04	−0.02	−0.07, 0.04	**0.36**	0.18, 0.54	0.13	0.02, 0.25	0.0004	−0.002, 0.00	0.001	−0.00001, 0.00
Proportion mediated	0.05	−4.54, 3.38	0.11	−1.89, 3.22	0.04	0.002, 0.13	0.03	−0.03, 0.28	0.02	−0.45, 0.72	0.04	−0.03, 0.37
*P* value-for-interaction	0.76		0.64		0.11		0.24		0.35		0.31	
Total no.	1,417		1,328		1,427		1,425		1,417		1,417	

*CI, confidence interval; OR, odds ratio; β, beta coefficient (mean difference)*.

*Bold indicates statistical significance after accounting for multiple comparisons using Bonferroni correction (p < 0.001)*.

† *Mediation analysis for the association of household income deprivation (exposure) with cardiovascular health (outcomes) via cortisol (mediator) adjusted for age, sex, and birthplace, with p-for-interaction between exposure and mediator*.

* *Mediation analysis for the association of household income deprivation (exposure) with self-reported CVD and diabetes (binary outcomes) via cortisol (continuous mediator) are fitted using the logistic regression for the outcome model and using linear regression for the mediator model, from which effect estimates with 95% CI are reported*.

### MR Study

A total of 1,562 Chinese adults were included, with more than half being women and born in Hong Kong (Table 1), as in the mediation analysis. The average age was 44.9 years (SD: 16.2). Mean cortisol was 8.2 ug/dl. Mean BMI was 23.5 kg/m^2^, mean body fat percentage was 27.3%, and mean systolic/diastolic blood pressure was 124/78 mmHg. The proportion with self-reported CVD was 3.6% and with self-reported diabetes was 4.7%.

Table 4 shows the associations of cortisol with CVD risk factors using the linear regression and MR. Higher cortisol was associated with lower BMI, the nominal association with lower body fat percentage, and higher systolic blood pressure was not evident after accounting for multiple comparisons. Using an internally weighted PRS for the MR analysis (*F*-statistic = 32.55–47.63 and *R*^2^ of 0.02–0.03), cortisol was unrelated to BMI, body fat percentage, and systolic and diastolic blood pressure. The linear regression and MR estimates did not differ based on the Hausman value of *p*.

**Table 4:** Adjusted^†^ association of cortisol with CVD risk factors (BMI, body fat percentage, and systolic and diastolic blood pressure) based on Mendelian randomization using genetically predicted log-transformed cortisol from internally weighted polygenic score and observational multivariable linear regression for 1,562 Hong Kong Chinese adults from Hong Kong's FAMILY Cohort.

**Outcomes**	**No**.	**Observational**		**Mendelian randomization**				
		**(multivariable linear regression)**		**(two-stage least square regression)**				
		**Mean**	**95% CI**	**Mean**	**95% CI**	**First-stage partial**	**First-stage**	**Hausman**
		**difference**		**difference**		***F*-statistic**	**R^**2**^**	***P*-value**
Body mass index	1,424	**−0.11**	−0.17, −0.05	−0.06	−0.76, 0.28	45.94	0.03	0.76
Body fat percentage	1,293	−0.12	−0.20, −0.04	−0.03	−1.83, 0.25	32.55	0.02	0.81
Systolic blood pressure	1,441	0.33	0.07, 0.60	0.41	−0.80, 4.45	47.63	0.03	0.93
Diastolic blood pressure	1,439	0.14	−0.03, 0.31	−0.002	−1.00, 1.00	47.63	0.03	0.78

*CI, confidence interval*.

*Bold indicates statistical significance after accounting for multiple comparisons using Bonferroni correction (p < 0.001)*.

† *Adjusted for age and sex*.

Table 5 shows the associations of cortisol with self-reported CVD and diabetes using the logistic regression and MR. Using an internally weighted PRS for the MR analysis (*F*-statistic = 41.6 and *R*^2^ of 0.03), cortisol was not associated with self-reported CVD or diabetes. The logistic regression and MR estimates did not differ as indicated by the Hausman value of *p*.

**Table 5:** Adjusted^†^ association of cortisol with self-reported CVD and diabetes based on Mendelian randomization using genetically predicted log-transformed cortisol from internally weighted polygenic score and observational multivariable Poisson regression for 1,562 Hong Kong Chinese adults from Hong Kong's FAMILY Cohort.

**Outcomes**	**No**.	**Observational**		**Mendelian randomization**				
		**(multivariable Poisson regression)**		**(multiplicative generalized methods of moment)**				
		**Incidence**	**95% CI**	**Risk**	**95% CI**	**First-stage partial**	**First-stage**	**Hausman**
		**rate ratio**		**ratio**		***F*-statistic**	**R^**2**^**	***P*-value**
Self-reported cardiovascular disease	1,388	1.03	0.95, 1.12	1.30	0.70, 2.40	41.26	0.03	0.47
Self-reported diabetes	1,388	1.06	0.99, 1.13	1.03	0.32, 3.37	41.26	0.03	0.97

*CI, confidence interval*.

*Bold indicates statistical significance after accounting for multiple comparisons using Bonferroni correction (p < 0.001)*.

† *Adjusted for age and sex*.

## Discussion

Considering both absolute and relative income at the household and neighborhood level among the Hong Kong Chinese adults, we found little clear or consistent associations of either absolute or relative income with poorer adult cardiovascular health in Hong Kong Chinese, with the mediating role of cortisol (as a biomarker of stress) remaining elusive.

Both greater relative household income deprivation and greater neighborhood income inequality were associated with lower BMI. In addition, the relative household income deprivation was associated with the higher systolic blood pressure, but not with CVD, diabetes, body fat percentage, or diastolic blood pressure. Neighborhood income inequality was not related with CVD or other risk factors. In addition, the absolute income at the household and neighborhood level was unrelated to CVD and its risk factors. These findings are inconsistent with the studies showing that the state-level income inequality was positively associated with CVD and its risk factors in some (2, 3, 6, 7), but not all (5, 8), Western countries. However, the state-level income inequality has not always been found to be associated with CVD risk factors in the West (4) and China (17, 19), especially, after adjusting for the individual absolute income. Our associations of relative household income deprivation with lower BMI but higher systolic blood pressure in Hong Kong Chinese adults are consistent with a previous Chinese study (19), but in that study the relative income deprivation was also associated with higher nutritional intake and smoking. Our mostly null findings for neighborhood income inequality suggest that the effects (if any) of income disparities across neighborhoods might be context dependent. That is, any deleterious effects of income deprivation (either absolute or relative) might be buffered in settings with the strong social infrastructure and resources. Hong Kong is an economically developed Chinese setting with extensive public transport, accessible and affordable public healthcare, free universal education, and a social safety net.

Further, our associations of relative household income deprivation with the higher systolic blood pressure, but with lower BMI, are counter-intuitive. To explore the potential mechanisms that could underlie any effects of the relative income deprivation on cardiovascular risk, in a subset of participants, we examined the role of cortisol. However, cortisol did not mediate the positive association of relative household income deprivation with systolic blood pressure after accounting for multiple comparisons. Indeed, we found little evidence of a direct effect of cortisol levels on CVD, suggesting that there was no role for cortisol to mediate any association between income variables and CVD. These discrepancies might also reflect different physiological drivers of BMI and blood pressure. Observationally cortisol was associated with lower BMI, but it was no longer associated with body fat percentage or systolic blood pressure after accounting for multiple comparisons, whereas using MR, cortisol was unrelated to CVD, diabetes, or other CVD risk factors in Chinese, possibly due to the small sample. Three recent MR studies have suggested that cortisol is positively associated with IHD in Western countries (31–33). However, in the two studies by Crawford et al., the positive estimate was found using IHD from the UK Biobank (33), but other estimates had CIs including the null using IHD from the CARDIoGRAMplusC4D (33) or the meta-analysis of the UK Biobank and CARDIoGRAMplusC4D (31), and in the other study by Pott et al., the sample was mainly patients with suspected or confirmed IHD (32), which is open to selection bias (34). Furthermore, our MR study in Westerners using more genetic variants for cortisol than those previous studies and found no association of cortisol with CVD and its risk factors (35). Replication using functionally relevant genetic variants for cortisol or conducting a meta-analysis of future MR studies on cortisol and CVD from China and elsewhere would help clarify the role of cortisol.

Several limitations are noted. First, the monthly average household income was reported in income categories rather than exact amounts. Using 29 income categories enabled granular ranking of income differences considering the willingness of participants to report. Second, household disposable income, consumption, possessions, or wealth could be a more direct measure than gross household income but this is more difficult to measure and standardize. Third, relative income deprivation is open to uncertainty as to the actual comparison group. Colleagues, relatives, or friends could be a more proximate comparison group, but we did not have such information. Fourth, household income and cardiovascular risk outcomes were measured at the same time. Longitudinal assessment would provide clearer temporality. This also limited the ability to control for potential confounders of the relationship, so all presented findings are descriptive in nature. Fifth, household income, and some health outcomes, e.g., CVD and diabetes were self-reported and hence are subject to recall bias. Under-reporting of household income and disease status is possible and might have attenuated the observed associations. Sixth, given this study focused on the overall association of income with cardiovascular health, we did not adjust for the traditional cardiovascular risk factors, e.g., smoking, physical inactivity, and other comorbidities in the observational analyses because they could be mediators rather than confounders. Seventh, candidate SNPs for cortisol in the Chinese were informed by GWAS in Westerners. Although we accounted for allele frequency in Chinese populations such that the SNPs had considerable genetic variation, the included SNPs may not all be relevant to cortisol among Hong Kong Chinese. Eighth, no genome-wide significant (*P* < 5 × 10^−8^) SNPs for cortisol in Chinese was identified, which is not unexpected given few have been found in Westerners. Larger, extensively genotyped GWAS for cortisol in Chinese in future would allow identification of more relevant SNPs for MR. Finally, we adjusted genetically predicted cortisol for age and sex but not for principal components because we used selected SNPs for cortisol not a full GWAS of the FAMILY Cohort.

In summary, relative household income deprivation was not consistently related in a cross-sectional analysis to cardiovascular health in Hong Kong Chinese, nor were neighborhood income inequality and absolute income. The relevance of income deprivation (either absolute or relative) seems to be context-specific and the psychosocial mechanism underlying relative income deprivation, including the role of cortisol, is likely complex and remains to be elucidated.

## Data Availability Statement

The data analyzed in this study is subject to the following licenses/restrictions: the data that support the findings of this study are available from the FAMILY Cohort in Hong Kong but restrictions apply to the availability of these data, which were used under license for the current study, and so are not publicly available. Data are however available from the authors upon reasonable request and with permission of the FAMILY Cohort. Requests to access these datasets should be directed to FAMILY Cohort, familyco@hku.hk.

## Ethics Statement

The studies involving human participants were reviewed and approved by University of Hong Kong/Hospital Authority Hong Kong West Cluster Institutional Review Board. The patients/participants provided their written informed consent to participate in this study.

## Author Contributions

MKK conceptualized ideas, performed the literature review, conducted data analysis, interpreted findings, and drafted the manuscript. IK, DR, MYN, and GML interpreted findings and revised drafts of the manuscript critically. CMS conceptualized ideas, directed analytic strategy, interpreted findings, revised drafts of the manuscript critically, supervised the study from conception to completion, and guarantor. MKK and CMS had full access to all the data (such as statistical reports and tables) in the study and can take responsibility for the integrity of the data and the accuracy of the data analysis. All authors contributed to the article and approved the submitted version.

## Funding

This study was supported by the Health and Medical Research Fund (HMRF) Research Fellowship Scheme, Food and Health Bureau, Hong Kong SAR Government (#02160107). This work is a sub-study of the FAMILY Cohort, with the establishment of the original cohort being funded by the Hong Kong Jockey Club Charities Trust from 2007 to 2014. The FAMILY Cohort Eye Biobank is part of the Chloe Ho Safeguarding Vision Initiative and was funded by the Jessie & George Ho Charitable Foundation.

## Conflict of Interest

The authors declare that the research was conducted in the absence of any commercial or financial relationships that could be construed as a potential conflict of interest.

## Publisher's Note

All claims expressed in this article are solely those of the authors and do not necessarily represent those of their affiliated organizations, or those of the publisher, the editors and the reviewers. Any product that may be evaluated in this article, or claim that may be made by its manufacturer, is not guaranteed or endorsed by the publisher.

## Abbreviations

CVD: cardiovascular disease
GWAS: genome-wide association study
IHD: ischemic heart disease
MR: Mendelian randomization
PGS: polygenic score
SNP: single nucleotide polymorphism.
